# The epistemological foundations of organicism: a reconstruction of Steiner’s *Grundlinien einer Erkenntnistheorie der Goetheschen Weltanschauung* (1886)

**DOI:** 10.1007/s40656-026-00745-2

**Published:** 2026-09-01

**Authors:** Christoph J. Hueck

**Affiliations:** Akanthos Academy Stuttgart, Zur Uhlandshöhe 10, 70188 Stuttgart, Germany

**Keywords:** Organicism, Epistemology, Rudolf Steiner, Goethean science, Participatory science, Philosophy of biology

## Abstract

Rudolf Steiner’s *Grundlinien einer Erkenntnistheorie der Goetheschen Weltanschauung* (Outline of an Epistemology of Goethe's Worldview, 1886) seeks to articulate the epistemological foundations of Goethe’s scientific method. Here I reconstruct the book with a particular focus on the cognition of organisms and read it in systematic relation to Kant, whose analysis of teleological judgment provides the clearest foil for Steiner’s project. The guiding question is under what epistemic conditions living beings can become objects of genuine knowledge rather than merely regulative judgment. I reconstruct the argument in five steps: (1) pure experience as a structureless manifold; (2) thinking as a self-transparent and law-governed activity within experience; (3) cognition as the reunification of perception and concept under empirical constraint; (4) the contrast between inorganic and organic cognition; and (5) the claim that organisms can be understood through Goethe’s concept of *Typus*, a developing method, and an intuitive power of judgment (*anschauende Urteilskraft*). Steiner’s reconstruction of Goethe thus yields a position that transforms Kant’s view of teleological judgment as merely regulative. A concluding section considers the relevance of this argument for current debates on organism-centred theoretical biology and the relation between subject and object in biological cognition. I suggest that Goethe’s method as interpreted by Steiner deserves renewed attention as a systematic attempt to articulate the epistemological conditions of a participatory science of the organic.

## Introduction: why read the *Grundlinien* today?

### The persistent problem of the organism

The question of how living organisms can become objects of genuine knowledge has been a recurring theme in philosophy since Aristotle. One of its clearest formulations is found in Kant’s *Critique of Judgment* (1790). Kant argued that the apparent purposefulness and self-organisation of an organism cannot be captured by the causal-mechanical categories that govern inorganic nature (AA V:372–376).^1^ We must judge organisms as if an idea of the whole were determining the formation of the parts—yet, according to Kant, we cannot claim to know that this is actually the case. For Kant, the teleological judgment remains merely regulative: a heuristic guide for research, not a constitutive principle of nature itself (McLaughlin, 1990).

Despite more than two centuries of progress in biological research, the epistemological problem identified by Kant has not been resolved; rather, if anything, it has become even more acute. The rise of molecular biology in the twentieth century initially suggested that organisms could, after all, be explained mechanistically—that the “mystery of life” would be revealed through the analysis of DNA, protein synthesis and biochemical metabolic pathways. However, it has become increasingly clear that the self-generating nature of the organism cannot be reduced to the properties of its molecular components (Nicholson, 2013; Woese, 2004). A holistic view of the organism has “returned” to biology—not as a relic of vitalism, but as a concept with which contemporary philosophy of biology must grapple (Nicholson, 2014). Likewise, the question of how evolution is to be understood has re-emerged in organism-centred form, particularly in debates surrounding the Extended Evolutionary Synthesis (Laland et al., 2015; Müller, 2017). Current philosophy of biology discusses these organismic aspects in terms of teleology (Breitenbach, 2009; Toepfer, 2012; Walsh, 2012), self-formation, normativity and function, autonomy and organismic agency (Di Paolo, 2005; Moreno, 2018; Sultan et al., 2021; Walsh, 2015), biological individuality and self-production (Fulda, 2023; Weber & Varela, 2002), the processual character of living beings (Meincke, 2023; Nicholson & Dupré, 2018), as well as the relation between subject and object in participatory approaches of organic cognition (Bortoft, 1996; Hueck, 2026; Rupik, 2024). However, as Wolfe, (2024) has shown, so-called “organistic” perspectives often tend to invoke Kant’s epistemological framework whilst uncritically linking it to ontological claims about the irreducible features of organisms—a conflation that leaves open the question of *how* organisms can be recognised, rather than merely establishing *that* they differ from machines.

So how can the living, self-generating form of an organism—its characteristic spatial organisation and its developmentally driven changes—become the object of genuine scientific understanding? In earlier works, I have argued that this question can be addressed through a method of cognition that follows the self-formation of the organism from within and intuitively grasps its holistic idea as it manifests itself in individual forms and states—and that such a method was developed by Johann Wolfgang von Goethe in his treatise on the *Metamorphosis of Plants* (1790) (Goethe, 1987; Hueck, 2025a, 2025b, 2025c).

Goethe, who was not only a poet, but also a distinguished scientist, reflected with considerable precision on the epistemological premises of his scientific practice (Hueck, 2025c), yet he never formulated a systematic theory of his method of knowledge. Rudolf Steiner (1861–1925), one of the first editors of Goethe’s scientific writings, recognised early on the distinctive nature of Goethe’s approach and its significance, particularly for the understanding of the organic (Steiner, 1987). Thus, the creation of a systematic epistemological foundation of Goethe’s worldview became the explicit aim of Steiner’s first philosophical work: the *Grundlinien einer Erkenntnistheorie der Goetheschen Weltanschauung*, published in 1886 (Steiner, 1886).

### Steiner’s *Grundlinien* as a philosophical project

The *Grundlinien* is a short but densely argued treatise—not a direct commentary on Goethe, but, as Steiner emphasised, an independent philosophical argument: a theory of knowledge that happens to coincide with the epistemological practice implied in Goethe’s scientific approach (Steiner, 1886).

Steiner wrote the *Grundlinien* at a time when Neo-Kantianism had become one of the dominant currents in German-speaking philosophy (Beiser, 2014) and reductionist natural science seemed to confirm the mechanistic program at every turn: Wöhler’s synthesis of urea, Darwin’s theory of natural selection, the rise of experimental physiology, and the physiological reductionism of Helmholtz and Du Bois-Reymond (Cahan, 2003). Against this backdrop, Steiner regarded the epistemological problem of organic knowledge as fundamentally unresolved.

The philosophical significance of Steiner’s project can best be formulated by reference to §77 of the *Critique of Judgment*, in which Kant introduces the thought experiment of an “intuitive understanding” (*intellectus archetypus*)—a method of cognition that would proceed from the whole to the parts and grasp the particular as a necessary expression of the general, rather than judging discursively by means of universal concepts. Such an understanding would not have to judge organisms *as if* they were purposeful; it would directly *see* their purposefulness. Kant emphasises, however, that this capacity is conceivable only as the opposite of our own, discursive understanding—not impossible, yet not our own (AA V:406–407).

Steiner’s project can therefore be understood as an attempt to respond simultaneously to two challenges: the Kantian restriction of organic knowledge to a purely regulative status, and the growing prestige of a mechanistic natural science that left no room for a genuinely immanent purposefulness. Against both, he advocates a position he termed “objective idealism” (Steiner, 1923): Thinking, properly understood, is not the imposition of subjective forms upon an alien content, but the self-revelation of the world’s own ideal structure within human consciousness. If this claim can be upheld, then the teleological unity of the organism need not remain something merely projected by us but can become an object of genuine knowledge.

### Scope and method of the present paper

This work is a philosophical reconstruction: it traces the line of argument in the *Grundlinien* through those phases which establish the epistemological prerequisites for the cognition of living organisms and examines each phase in dialogue with the corresponding Kantian positions. The aim is neither a historical commentary nor a hagiography, but a systematic presentation of how Steiner’s account of the Goethean mode of cognition overcomes the Kantian limitations on the cognition of organic beings.^2^ The claim is therefore that Steiner’s reconstruction of Goethe can be read as an epistemological model for contemporary organism-centred thought in theoretical biology.

The reconstruction is therefore selective: It does not deal in detail with the introductory *Preliminary Questions* (Chapters 1–3), in which Steiner clarifies his starting point, situates Goethe’s science in relation to Schiller, and formulates the task of his epistemology. These chapters are indispensable for the book’s programmatic self-understanding, but do not yet contain the core argument with which Steiner seeks to ground the knowledge of organisms. Nor does the reconstruction offer a systematic treatment of the final chapters (17–21), in which Steiner extends his epistemological framework to the fields of the humanities, freedom and art. These chapters demonstrate the far-reaching nature of Steiner’s project, yet the present argument is limited to those parts of the book that prepare for and justify the understanding of the organic.

The paper is structured as follows: Sect. 2 reconstructs Steiner’s starting point: the concept of “pure experience” and its relationship to Kant’s phenomenal world. Section 3 analyses the central thesis that thinking is a “higher experience within experience”, the laws of which are self-transparent. Section 4 shows how Steiner reconceptualises the act of cognition as a reunification of perception and concept, and how this enables him to reject both subjective idealism and naive realism. Section 5 briefly turns to inorganic nature in order to clarify the lawful relationships governing it, as well as the limit at which a different kind of cognition becomes necessary. Section 6 reconstructs the argument concerning organic nature: the concept of the type (*Typus*), the distinction between “demonstrative” and “developing” methods, and the claim that the knowledge of organisms requires an “intuitive power of judgement” (*anschauende Urteilskraft*). Section 7 offers a concluding assessment and discusses the relevance of the argument for current organism-centred debates in theoretical biology and philosophy of biology.

## Pure experience and the rejection of transcendental subjectivism

### The demand for a presuppositionless starting point

Every theory of knowledge must begin somewhere, and the choice of starting point is never arbitrary. Kant’s *Critique of Pure Reason* begins with the assertion that “all our knowledge proceeds from experience” (AA III:B1), but he immediately qualifies this by distinguishing between what is *given* through sensory perception and what is *contributed* by the understanding, thereby separating the subjective forms of knowledge (space, time, the categories) from a reality that remains fundamentally inaccessible in itself (Allison, 2004, pp. 20–49; Guyer, 1987, pp. 11–24). The Neo-Kantians of Steiner’s time regarded this distinction as settled: Otto Liebmann’s famous dictum—“we must return to Kant” (Liebmann, 1865, p. 110)—had become a battle cry, and with it the conviction that the primary task of epistemology was to acknowledge the constitutive limits of human knowledge (Beiser, 2014).

Steiner’s first step in the *Grundlinien* consists in rejecting precisely this starting point: Epistemology, he emphasises, must begin without any preconceptions about the nature of experience or the limits of knowledge. Any assertion as to whether the world we encounter is “purely subjective” or “objectively real”, whether it is appearance or thing-in-itself, is already a *result* of thought—a judgement that must be justified rather than accepted as a given. To place such a judgement at the outset of the investigation means sidestepping precisely the question that the investigation is actually intended to answer (Steiner, 1886).

This methodological requirement has a distinctly Goethean character: just as Goethe sought to approach natural phenomena without preconceived theoretical commitments, Steiner demands that the epistemologist approach experience without preconceived metaphysical commitments—starting from what is simply *given*, prior to any theoretical determination of its nature.^3^

### Pure experience as structureless manifold

What, then, is simply given? To answer this question, Steiner introduces the concept of *pure experience*: reality as it presents itself when we encounter it “with a complete relinquishment of our self” (*mit vollständiger Entäußerung unseres Selbstes*), before thought intervenes to organise, connect or interpret it (Steiner, 1886).

Understood in this way, pure experience differs radically from the structured, ordered world in which we usually live. It is, Steiner argues, “a mere succession in space and sequence in time, a collection of entirely unconnected details” (Steiner, 1886). No object in this diversity has greater significance than any other: an animal’s rudimentary organ counts for just as much as its most vital part; Caesar’s significance for history is no greater than that of one of his soldiers. Pure experience is, in Steiner’s vivid conception, “a perfectly flat surface” on which no point rises above another—until the “spark of thought” strikes and creates elevations and depressions, distinctions and connections (Steiner, 1886).

The concept of pure experience is not merely descriptive, but *methodological*. Steiner does not claim that we ever actually *inhabit* such a state. In the *Grundlinien*, his description of pure experience as a “mere spatial juxtaposition and temporal succession” might suggest that spatial and temporal structure is already present in the given. Yet Steiner clarified this point in his later epistemological writings. In *Wahrheit und Wissenschaft* (1892), he characterises the given (*das unmittelbar Gegebene*) more strictly as entirely indeterminate: a “coherent yet not even broken down into individual details world-picture, in which nothing is distinguished from anything else, nothing stands in relation to anything else, nothing appears to be determined by anything else” (Steiner, 1892). In *Die Philosophie der Freiheit* (1894) he is even more precise: The pure content of observation, which precedes all thinking, is nothing other than “the mere incoherent collection of sensory objects: colours, sounds, pressure, warmth, sensations of taste and smell; also feelings of pleasure and displeasure” (Steiner, 1894).^4^ What pure experience lacks, therefore, are not merely *intelligible connections*, but any conceptual structure whatsoever—including the individuation of objects, their spatial and temporal order, and every relationship that makes the world comprehensible. All this, Steiner argues, is contributed by what he calls “thinking.” Pure experience as such contains no explanation—but neither, taken on its own, does it pose a question. It is the thinking observer who, confronted with the unstructured given, raises the question that science must answer.

### Steiner’s *reine Erfahrung* and Kant’s manifold of intuition

Steiner’s concept of pure experience invites comparison with Kant’s “manifold of intuitions”—that raw material which, according to the *Critique of Pure Reason*, is given through sensory perception before being synthesised by the understanding (Kant, 1787). The two positions exhibit a structural parallel—both proceed from an initial, unorganised given that must be processed by thought in order to produce knowledge—yet their similarities and differences must be set out with care.

In the Transcendental Aesthetics, Kant argues that space and time are not properties of things-in-themselves, but a priori* forms of sensibility*: conditions which the subject contributes to every act of perception (Kant, 1787). His argument proceeds from the observation that we can conceive of space and time without objects, but not of objects without space and time; therefore, space and time cannot be derived from experience, but must be presupposed by it (Guyer, 1987, pp. 345–350). However, for experience to arise, the forms of sensibility require *matter*, that is, sensory content (colours, sounds, tactile qualities); for Kant, this presupposes that sensibility is affected by something given independently of those forms, “the objects in themselves” (Kant, 1787). Kant thus distinguishes within every intuition a *formal* element (contributed by the subject) and a *material* element (originating from outside)—and the decisive Kantian step consists in explaining that, since the forms of space and time are subjective contributions, the entire phenomenal world belongs solely to the realm of *appearances*, not to things as they are in themselves.

Steiner’s description of the given as a collection of mere sensory qualities—colours, sounds, pressure, warmth—comes remarkably close to what Kant calls the *matter* of sensation. Both thinkers agree that the raw starting point of cognition is a multiplicity of unconnected sensory impressions, which as such do not yet constitute knowledge. What Steiner calls the “structureless manifold” and Kant calls the “manifold of intuition” occupy the same systematic position: they are the material that must be processed through cognitive activity before experience in the full sense can arise.

The decisive difference concerns not the character of the starting point, but the manner in which cognition subsequently takes up and elaborates the immediately given. For Kant, the sensory manifold becomes coherent experience only through a synthesis carried out under a priori forms contributed by the subject: space and time as forms of sensibility, and the categories as forms of the understanding (Kant, 1787). On this basis, the entire structured world of experience—everything we perceive as ordered in space and time—belongs to the realm of phenomena, whose form is conditioned by the subject, and not to things as they are in themselves. For Steiner, however, the question of whether the forms that thought introduces into the given are subjective or objective cannot be settled in advance. Since every judgement on this matter is itself an act of thinking, the question can only be answered after the nature of thinking itself and its relation to experience has been examined (GA 2:37; GA 2:56). This is not a refusal to engage with Kant’s question, but an insistence that it must not be prejudged.

Kant’s own framework therefore creates a further problem: if the forms that structure experience are subjective contributions, then the sensible content of experience must nevertheless come from somewhere; for Kant, it presupposes that sensibility is affected by something given independently of those forms, something thought as the thing in itself. However, if the thing in itself is fundamentally unknowable, the claim that it nevertheless affects sensibility becomes deeply problematic, as Kant’s contemporaries, from Jacobi to Schulze, were quick to point out (cf. Allison, 2004, pp. 64–65).

Steiner, on the other hand, does not start from a metaphysical division that must then be overcome, but from the undivided given—and asks: What does thinking reveal, when we examine it, about the nature of this given?

### The critique of transcendental subjectivism

This leads Steiner to one of his sharpest criticisms of the Kantian and Neo-Kantian tradition. In Chapter 6 of *Grundlinien* (“Correction of a Misconception of Total Experience”), he argues that the view that the entire world of perception is nothing more than “a representation within our individual [i.e., subjective, C.H.] consciousness”, is not a fact of experience but a *theoretical judgement*—a “prejudice” that has no place at the foundation of epistemology (GA 2:36–39).

Steiner’s argument proceeds in two steps. First, he observes that nothing in the immediate nature of experience justifies the label “subjective”. A tree, a table, a colour—none of these things possesses a characteristic that would lead an impartial observer to describe them as “mere representations”.^5^ The fact that we regard the world of perception as subjective is not the result of experience itself, but of a differentiated theoretical reflection, particularly on the physiological conditions of perception (GA 2:36–37).^6^

Second, and more fundamentally, Steiner argues that the Kantian approach is incompatible with its own premises. Steiner cites Johannes Volkelt, who in *Erfahrung und Denken* (1886) had established what he called the “insurmountability of consciousness”: “It is simply impossible for me … to leap beyond the limits of my consciousness, to leave my consciousness” (Volkelt, 1886, p. 59). From this, Volkelt drew the radical conclusion that “I never reach the things as such”—all acts of cognition are “inseparably bound to the cognising, individual consciousness” and “utterly incapable of reaching beyond the realm of the individual and grasping or entering the realm of external reality” (Volkelt, 1886, p. 59; cf. GA 2:37).

Steiner’s objection is directed against the logical structure of this argument. If pure experience, as Volkelt himself had rigorously demonstrated, is a manifold of unconnected particulars without intrinsic relations (Volkelt, 1879, pp. 168–169), then the *cognitive subject* stands within this manifold just as disconnected from the rest as any other element. The perceiving subject is merely another particular within the manifold GA 2:38. To single it out and declare the entire manifold to be “its” representation is to introduce a relationship—the relationship between representation and the representer—which pure experience does not contain. Volkelt, whom Steiner regards as the most rigorous epistemologist of his time, is thus guilty of precisely the inconsistency he sought to avoid: Having established that pure experience contains no relationships, he nevertheless introduces the most consequential relationship of all—that between subject and object—even before the investigation of thought has begun. In doing so, “he had to be unfaithful to his own principle” (GA 2:38).

This criticism has direct implications for the problem of organic cognition that underlies the present study. If the Kantian framework declares every experience to be subjective from the outset, then the question of whether the functional unity of the organism “actually exists” is nipped in the bud before it is even posed. Under such a judgement, the formative principle of the organism can never be more than a useful fiction. Steiner’s rejection of transcendental subjectivism is thus not merely an academic disagreement over method; it is the prerequisite for any theory of knowledge that claims to offer genuine access to the living world.

### The question that emerges from pure experience

Having clarified what pure experience is and what it is not, Steiner turns to the question that will shape the rest of the *Grundlinien*: “Is the form in which experience presents itself something grounded in the nature of things? Is it a property of reality?” (GA 2:41).

If the disconnectedness of pure experience reflects the true nature of reality, then science can be nothing other than the cataloguing of isolated particulars. If, on the other hand, this disconnectedness is merely the “husk” (*Hülle*) (GA 2:42) of a deeper unity, then the means of penetrating to this unity must be sought *within experience itself*. This qualification shapes the following argument. Since any recourse to principles outside of experience—be they a priori forms, metaphysical causes or transcendent reasons—would violate the requirement of presuppositionlessness established in Sect. 2.1, the element that introduces lawful connections into the structureless manifold cannot be posited from outside of experience. It must itself be encountered within experience, and indeed *as* experience—something we do not postulate, but *find*, and which already bears within itself those intelligible connections that are lacking in the rest of experience. As Steiner puts it: “If we are to gain in thinking a means of penetrating more deeply into the world, then thinking must itself first become experience” (GA 2:29–30).

From a Kantian or analytical perspective, one might object that grounding epistemology in experience is fundamentally misguided: experience, precisely because it is pre-conceptual, cannot serve as a foundation for knowledge. This concern has been articulated as a critique of the “Myth of the Given” (McDowell, 2003; Sellars, 1997): no pre-conceptual content of experience can function as a *reason* for belief.^7^ However, Steiner *does not* claim that pre-conceptual experience yields knowledge—his characterisation of pure experience as unstructured negates precisely that. His claim is methodological: thinking must first be *identified as a phenomenon*—encountered within experience—before its nature and epistemic status can be determined. The alternative—to start with a theory about what thinking can achieve—would entail precisely the kind of untested presupposition that a critical epistemology must reject.

Steiner’s candidate for the sought-after element can now be named: it is thinking—not thinking as a capacity *presupposed* by epistemology, but thinking as a datum *encountered* within the manifold, a “higher experience within experience”, as Steiner will call it. Whether thinking actually fulfils these requirements—whether it contains within itself those intelligible connections that are lacking in the rest of experience—is the question to which the *Grundlinien* now turn: the transition from the unstructured “flat surface” of pure experience to the landscape of intelligible connections.

This transition also locates Steiner within the post-Kantian attempt to overcome the merely subjective status of thought (cf. Beiser, 2009). Steiner explicitly acknowledges that the insight into the “inner solidity and perfection of thinking” appears most clearly in Hegel’s system (GA 2:50–51). Yet he argues that Hegel did not make sufficiently clear that “the field of thought is solely human consciousness” and that this does not make the world of thought lose anything of its objectivity (GA 2:51). The decisive step in the *Grundlinien* is therefore not to begin, as Hegel does, with the concept, but to identify thinking itself as an experience and then ask what this experience reveals about its own content and validity.

## Thinking as higher experience within experience

### The encounter with thinking

In Sect. 2, it was established that the element which introduces comprehensible connections into the unstructured manifold of pure experience must itself be encountered *as* experience—found within what is given, not postulated from outside. In chapter 8 of *Grundlinien* (“Thinking as Higher Experience within Experience” [GA 2:43–48]), Steiner argues that thinking possesses precisely this character: It differs fundamentally from every other content of experience in a way which, if properly understood, resolves the epistemological impasse.

When we perceive a colour, hear a tone or feel warmth, we encounter a *finished product*—a content that is simply *there*, without revealing how it came into being. The red of the rose presents itself as a given; the conditions of its being given are not provided in the act of perception (GA 2:46–47). There is an unbridged gap between the given content and the process of its coming into being. Steiner even extends this contrast to other events of mental life, which likewise confront us as something given rather than as a process whose genesis we transparently accompany.

In thinking, however, the situation is qualitatively different. When I trace a chain of conclusions or articulate the connection between a law and the phenomena it governs, I am not merely a witness to a result whose origin remains hidden from me; I *perform* an activity, every step of which falls within my field of experience (GA 2:47). In thinking, production and product are not separated from one another, as is the case in every other sphere of experience. This makes thinking a “higher experience within experience”: unlike any other experience, it reveals its own genesis.

### The self-transparency of thinking

The claim that thinking reveals its own genesis requires more precise articulation: self-transparency (*Durchsichtigkeit*), as Steiner understands it, has two inseparable dimensions.

The first concerns *activity*: when I think, I am aware of myself as thinking—I follow the movement from one determination to the next, I direct the process, I recognise the transitions as my own performance.^8^ This awareness is not a retrospective reflection appended to a completed thought; it is part of the act of thinking whilst it is being carried out. Thinking that proceeds without any awareness of itself as thinking is not yet thinking in the proper sense—it is the involuntary flow of associations and habitual connections, which Steiner carefully distinguishes from thinking proper (GA 2:47).^9^

The second concerns the *content*: when I think through Pythagoras’ theorem—or follow a line of reasoning step by step—the necessity of the connection is not obscure to me. I do not merely take note of a fact; I *see why it must be so*. The inner necessity that connects the elements of thinking is given *in and with* the thinking itself. In this sense, the content of thinking is self-explanatory: its comprehensibility does not depend on a further act of explanation but is intrinsic to it (GA 2:43–44).

These two dimensions together—transparency of activity and transparency of content—constitute what Steiner calls the *Durchsichtigkeit* of thinking. Unlike every other sphere of experience, thinking does not leave the conditions of its own emergence hidden from the subject. Its activity and its content are given together in one and the same act. There is, so to speak, no hidden “back side” of thinking that remains inaccessible to the thinker.^10^

### Self-givenness and the objectivity of thought-content

From the self-transparency of thinking, Steiner derives a consequence that goes beyond Kant’s restriction of thought to subjective conditions. If the content of thinking is completely transparent—if nothing about its origin remains hidden from the thinker—then there is, in principle, no room for the suspicion that thinking might distort the content it produces. Let us consider once more the example of Pythagoras’ theorem. When I think it through, I do not merely accept the theorem as true; I follow the proof step by step and see why the conclusion must hold. If my thinking were to distort the content (just as coloured glasses distort the colours of objects), this distortion would have to occur somewhere in the chain of argumentation—and since every link in this chain is transparent to me, I would be able to recognise it.

But Steiner goes further. He argues that the content of thinking is not merely transparent, but *self-given* (*selbstgegeben*): it arises from the nature of thinking itself, not from the empirical characteristics of the individual thinker. Pythagoras’ theorem, for example, is determined by relationships inherent in the thought itself, and not by the psychological or physiological state of the thinker; the same content reveals itself to every thinker who follows the same proof, regardless of their physical constitution or cultural context. This universality is not imposed from without; it is discovered from within, as a property of the content of thought itself (GA 2:49).

The point is not merely that truth is universal, but that the source of this universality lies in thinking itself rather than in the thinker. Steiner expresses this with the utmost precision:

We do not produce the content of a thought in such a way that we determine, in the process of its production, which connections our thoughts must form. We merely provide the occasional cause (*Gelegenheitsursache*) for the content of the thought to unfold *in accordance with its own nature*. We take thought *a* and thought *b* and provide them with the opportunity to enter into a lawful connection by bringing them into interaction with one another. It is not our subjective organisation that *determines* this relationship between *a* and *b* in a certain way, but the content of *a* and *b* itself is the *sole determining factor*. We have not the slightest influence over the fact that *a* relates to *b* in a specific way and not otherwise. *Our mind comprehends the composition of the masses of thought solely in accordance with their content.* We thus fulfil, in thinking, the principle of experience in its most radical form. (GA 2:49).

This passage reverses the expected relationship between subject and content: the thinker does not impose a form on thoughts but provides the “occasional cause” so that thoughts may unfold their own regularity. Furthermore, Steiner expressly asserts that in thinking we fulfil “the principle of experience in its most rigorous form” (*das Erfahrungsprinzip in seiner schroffsten Form*) (GA 2:49). In the *Einleitungen zu Goethes naturwissenschaftlichen Schriften* (GA 1), Steiner formulates this point particularly succinctly: “the idea, which is the most objective, appears to us at the same time as the most subjective” (Steiner, 1987).

Steiner compresses this point in a single sentence: “We must imagine two things: first, that we bring the ideal world into appearance through our activity, and at the same time, that what we actively call into being rests on its own laws” (GA 2:52). We are, Steiner concedes, accustomed to regarding phenomena as things that we observe *passively*. “But that is not an unconditional prerequisite. However unfamiliar the idea may be that we ourselves actively bring objective content into manifestation—that we, in other words, do not merely perceive a phenomenon, but simultaneously bring it into being: it is not an inadmissible notion” (*sie ist keine unstatthafte*) (GA 2:52). This is the crux: thinking is the only instance in which the subject is actively involved in the production of the phenomenon, and yet the content of what appears is not determined by the subject, but by its own inner laws.

This dual assertion—that the subject *actively generates* the appearance of thought-content, and that this content nevertheless *determines itself* according to its own laws—forms the systematic core of the *Grundlinien*.

Steiner’s position can now be located with some precision on the map of post-Kantian philosophy (cf. Beiser, 2009). Against *Kant*, he holds that the content of thinking is objective and not merely a subjective form imposed upon an unknowable given. Against *Fichte*, he holds that the content is not produced by the ‘I’ but unfolds according to its own laws—the thinker is indispensable for the *appearance* of the content, but dispensable for its *determination*. With *Hegel*, he shares the conviction that thinking is objectively valid, yet insists that this validity must be grounded in the *experience* of thinking and not through dialectical deduction. Steiner later formulates the difference sharply: “I have designated thinking as my starting point, and not concepts and ideas, which are first gained through thinking”; Hegel, by contrast, “sets the concept as the first and original” (Steiner, 1894). The result is a position that might be described as *experiential objective idealism*: the ideal content is objective (contrary to Kant), self-given and not produced by the ‘I’ (contrary to Fichte) and is encountered as experience rather than deduced as a necessity (contrary to Hegel).^11^

### Thinking and the Kantian categories

The implications of Steiner’s position become clearer when contrasted with Kant’s doctrine of the categories. In the *Critique of Pure Reason*, Kant identifies twelve fundamental concepts of the understanding—substance, causality, reciprocity and others—which he treats as a priori* conditions* of possible experience (Kant, 1787; cf. Allison, 2004, p. 159 ff.). They are not derived from experience but are the forms through which the understanding synthesises the raw manifold of intuition into a coherent, objective experience; without them, experience would remain a “rhapsody of perceptions” without any lawful connection (Kant, 1787). Precisely because the categories are conditions under which objects can be recognised by us at all, their validity, for Kant, extends to phenomena, but not to things as they are in themselves (Kant, 1787; cf. Guyer, 1987, pp. 157–161).

From a Kantian perspective, therefore, the self-transparency of thinking outlined in Sects. 3.1–3.3 is not yet sufficient to demonstrate its objectivity: even if we can trace every step of a line of thought, the forms that make such insight possible might themselves be subjective conditions, and to use these forms to confirm their own objective status would be circular. The objection arising from Steiner’s analysis, however, is that this conclusion presupposes an assimilation of thinking to perception, which his analysis has already called into question. In perception, the conditions under which a content appears are hidden from the perceiver; this makes the suspicion of subjectivity understandable. In thinking, by contrast, content and genesis are given together. When I think through Pythagoras’ theorem, the necessity of the connection is not perceived as the result of a hidden faculty, but as something that follows from the content of the thought itself.^12^

The question, then, is not whether thinking imparts lawful relationships to the given—Steiner agrees that this is the case—but what *status* these relationships possess. For Kant, they are subjective conditions under which alone an object can appear to us. For Steiner, however, the relevant question is whether Kant’s conclusion regarding subjectivity still holds true even when the origin of the connection is not hidden from the thinker. The thinker’s activity provides the *occasional cause* for the relationship to become visible; however, since this relationship becomes visible precisely through the thinker’s own activity, its emergence is transparent and its content is not mediated by a hidden subjective factor (GA 2:50). The argument therefore does not simply assert objectivity against Kant’s subjectivism; rather, it identifies the specific condition under which the Kantian conclusion would follow—namely, the opacity of the cognitive process to itself—and argues that this condition does not hold in the case of thinking.^13^

### Thinking investigating itself and the coincidence of knower and known

A further question concerns not the objectivity of thinking, but the legitimacy of examining thinking by means of itself. If thinking is the instrument with which we explore the world, and thinking is at the same time the object under investigation, does the investigation not then presuppose what it purports to establish? This objection takes on particular significance in the context of Steiner’s argument, as he insists that thinking must be investigated *as experience*—that is, through precisely that capacity whose nature is under debate.^14^

Steiner addresses this directly. The exploration of thinking through thinking does not constitute a problematic circle, since thinking is the only realm in which the *instrument* and the *object* are identical. When thinking turns in on itself, it does not face an alien object; it encounters *itself* in precisely that act through which it is recognised. The apparent “circle” is therefore not a shortcoming, but a unique epistemological situation in which the knower and the known coincide (GA 2:48; Steiner, 1894).

This coincidence is more than the resolution of a reflexive difficulty. It establishes the structural pattern that will prove significant for Steiner’s later account of organisms: when reflecting on thinking, cognitive activity produces precisely the content that it recognises. When Steiner later argues that the cognition of organisms requires a similar coincidence—that the cogniser must internally *produce* the formative principle (*Typus*) of the organism in order to recognise it (Sect. 6)—he extends the cognitive structure to the realm of organic nature, the possibility of which has already been demonstrated within the realm of thinking itself.

Yet an important question remains: *Where does the content of thinking come from?* We have not yet clarified the *relationship* between this self-contained ideal content and the world we encounter through perception. Is the content of thought a self-contained ideal realm, complete in itself but having no connection to sensory data? Or does it belong to the *same reality* that presents itself from another angle as perception? In other words: are the intelligible connections revealed by thinking also the intelligible connections *of things*?^15^

This question cannot be answered solely within the framework of an analysis of thinking. It requires an account of how thinking and perception *meet*—how the ideal content revealed through thinking relates to the sensually given content of perception. Steiner’s answer, which he develops in chapters 11–13 of *Grundlinien*, is that cognition is the *reunification* of two aspects of a single reality that have been separated only by the organisation of the cognising subject: perception provides the outer, sensory side; thinking reveals the inner, ideal side; and the act of cognition brings them together. It is only through this reunification that the ideal content of thinking reveals itself as the ideal content *of things*—and only then is Kant’s restriction on teleological judgement truly overcome. This is the subject of Sect. 4.

## Cognition as reunification

### The separation of reality into two domains

The analysis of Sects. 2 and 3 has raised a new problem. Perception presents a multitude of unrelated details; thinking reveals an internally structured ideal content. But the relationship between the two has not yet been established. Man thus stands, in Steiner’s words, as a “citizen of two worlds”: “the world of the senses, which presses upon him from below, the world of thought, which illuminates from above” (*die eine von unten an ihn herandringend, die andere von oben leuchtend*) (GA 2:79).

For Steiner, this duality is not a metaphysical division of reality itself, but a consequence of the organisation of the knowing subject. Sensory perception and thinking provide their respective contents separately from one another, and it is precisely this separation that gives rise to the epistemological problem: “Reality has divided itself into two realms for us: into experience and into thinking” (GA 2:76).

### Perception and thinking as the two sides of reality

A characteristic error of modern science, Steiner argues in Chapter 11 of *Grundlinien*, is to treat sensory perception as something “self-contained and complete” (GA 2:62). Yet in doing so, half of reality is confused with the whole: “Sensory perception provides only one side of reality. The other side is the thinking apprehension of the world” (GA 2:63).

Thinking reveals the lawful connection that perception alone does not show. If this connection is experienced not as a subjective imposition but as arising from the content of thinking itself, then thinking must be understood as the organ through which the ideal structure of reality comes to light.^16^ What Steiner had already formulated in the *Einleitungen* now receives its systematic justification: “Empirical reality must in general have two sides: one according to which it is particular, individual, and another according to which it is ideal and universal” (Steiner, 1987). The ideal and the sensible are therefore not two worlds, but two sides of one and the same reality.

This does not negate the difference between the two. Thinking provides a lawful determination; perception supplies that specificity which thinking cannot provide on its own. Here, Steiner’s position approaches Kant’s statement that thoughts without content are empty and intuitions without concepts are blind (Kant, 1787), though only to a certain extent. For Steiner, however, the world of thought is not empty without perception: it is internally structured and self-determined. What perception adds is not content to an otherwise empty form, but concreteness to an already lawfully structured ideal content. The difference is crucial, for it is precisely this inner productivity of thinking that prevents the world of concepts from being the “empty schema” that Kant took it to be.

### The act of cognition: *Wahrnehmungsurteil* and *Wiedervereinigung*

The act of cognition is the moment in which the two sides of reality come together. Steiner describes the process with phenomenological precision:

We confront a concrete perception. It stands before us like a riddle. Within us, the drive makes itself felt to investigate its true *what*, its essence, which it does not itself express. This drive is nothing other than a concept working itself up out of the darkness of our consciousness. We hold fast to this concept while the sense-perception runs in parallel with this thinking process. The mute perception suddenly speaks a language we understand: we recognize that the concept we have grasped is the sought-after essence of the perception. (GA 2:64).

Steiner calls the judgment that unites a perception with its concept a “perceptual judgment” (*Wahrnehmungsurteil*). It differs fundamentally from a purely conceptual judgment (which connects two concepts without reference to perception). In a perceptual judgment, the subject is a perception and the predicate a concept: “This particular animal that I have before me *is* a dog” (GA 2:65). The perception is thereby “inserted into my thought-system at a determinate position” (GA 2:65)—it ceases to be a mere disconnected particular and reveals its place within the intelligible order of the world.

In *Wahrheit und Wissenschaft* (1892), Steiner sets out the process of cognition with systematic clarity, whilst also taking into account the possibility of error: if we wish to recognise something beyond thinking itself, he argues, thinking must approach a given reality and, drawing it out of its chaotic interweaving, place it within a systematic relationship within the world-picture. In doing so, thinking first isolates individual elements from the continuous whole of the given, then relates them to one another in accordance with the forms it has produced, and finally grasps what emerges from the established relationship. If the content of the world has nothing to reveal through the established relationship, then the attempt at thinking has failed and must be replaced by another. It is crucial that thinking does not arbitrarily determine the content from its own perspective: by relating two separate parts of the content of the world, it *waits* to see what emerges from this relationship itself. Only this result counts as knowledge (Steiner, 1892).

This point deserves special emphasis. Steiner’s conception of knowledge is not that of a self-contained idealistic system in which thought unfolds without empirical constraints. The connections established through thought must stand up to scrutiny in relation to perception: it is an empirical claim to the content of the world, not a purely internal movement of concepts. In this sense, the *perceptual judgement* involves both a test and the possibility of failure. A conceptual connection is only considered cognitively valid if, once brought into the relevant relationship, the content of perception actually reveals the lawful unity sought by the thinker; if it does not, the attempt has failed. Steiner does not, however, develop explicit criteria for such success or failure. What his account implies is that the validity of a cognitive claim depends on whether the relation established in thought enables the content of perception to be understood as a lawful whole, rather than as a merely subjective construction.^17^

The philosophical crux lies in Steiner’s characterisation of what occurs in this process. Cognition is not the *imposition* of a concept upon a perception from the outside (as Kant’s model of synthesis suggests), but rather the *reunification* of two aspects of a single reality, which were merely separated from one another by the organisation of the cognising subject. As outlined in Sect. 4.1, this separation is not a feature of reality, but a consequence of the fact that human beings apprehend the ideal and the sensory through two different faculties. Cognition reverses this separation: it brings together what was never separated in the thing itself. The separation of perception and concept is, in Steiner’s words, a necessary *stage* of cognition, not its endpoint. Cognition in the true sense arises when thinking provides the ideal content—the meaning and the context—that perception lacks, thereby restoring the unity that the thing possessed before the dual organisation of the perceiving subject divided it into a sensory and an ideal aspect (GA2:77).

### The contrast with Kant’s synthesis

Steiner’s conception of cognition as *reunification* stands in direct contrast to Kant’s theory of synthesis. For Kant, the act of cognition consists in the understanding *subsuming* the manifold of intuition under its a priori concepts (the categories). The understanding does not discover a unity that is already present in the object as such but introduces the unity under which alone an object can appear to us (Kant, 1787; Allison, 2004, p. 159 ff.). The consequence is obvious: every cognitive unity is a subjective unity, and nothing we know about objects can ever be more than a description of how objects appear *to us*.

Steiner’s opposing view is that cognition does not project unity onto perception but *restores* a unity that was never absent from the thing itself. The concept is not brought into perception from outside; it is the meaning and lawful connection of the perceptions themselves, separated from them only by the organisation of the knowing subject.^18^ Steiner’s model is thus neither subjective idealism (which denies the independent reality of the perceived world) nor naive realism (which regards perception as the entirety of reality), but rather what he later termed “objective idealism” (Steiner, 1923): the conviction that thinking by no means distorts reality, but rather *completes* it—that the ideal content revealed by thinking is the objective meaning of things, not a subjective addition to them.^19^

Steiner takes this idea a step further. If thinking were merely a subjective addition to an already complete reality, it would be superfluous—“one would truly be unable to recognise what necessity there might be at all for rising above the sensory world” (GA 2:58). Yet thinking reveals that aspect of reality which can appear *only* to thinking: the meaning and the lawful context of the sensory phenomena which perception provides, but which it cannot articulate of its own accord. The mind, Steiner argues, “is just as much an organ of perception as the eye and the ear. The thought relates to our spirit no differently than light relates to the eye or sound to the ear” (GA 2:77).^20^

### The inner coherence of the thought-world and the origin of Ideas

The argument so far has shown that cognition reunites perception and concept. Yet a further step is required before the full implications for the problem of organic cognition can be drawn. In Chapter 10 of *Grundlinien* (“The Inner Nature of Thinking”), Steiner argues that the concepts revealed through thinking do not stand in isolation from one another. They form a coherent, organically connected totality. Every concept has its place within a unified world of thought; a concept that stands apart from the rest is, for Steiner, “something unnatural, an untruth” (*eine Unnatürlichkeit, eine Unwahrheit*) (GA 2:57).

Steiner illustrates this with a characteristic example: “In our consciousness we find the concept of ‘organism’; when we survey our world of ideas, we encounter a second one: ‘law-governed development, growth’. It immediately becomes clear that these two concepts belong together, that they merely represent two sides of one and the same thing” (GA 2:56). The connection between “organism” and “law-governed development” is not imposed by the thinker; it is found in the content of the concepts themselves. Every concept, when fully thought through, points beyond itself to other concepts with which it forms a higher unity.^21^

This inner interconnectedness of the world of thought has an important consequence. When thinking traces the connections between its concepts, higher units emerge—units that unite several individual concepts into a single, comprehensive determination. These higher units are what philosophy, since Plato, has referred to as *Ideas*. In this sense, an idea is not a vague generality or a rigid abstract universal, but a dynamically articulated unity in which a multitude of concepts belong together and find their place within a whole. Its unity is therefore not fixed once and for all: depending on the wider context in which it is understood, it can be further specified, expanded or interpreted differently.^22^

The question, then, is what epistemic status these ideas possess. On this point, Steiner and Kant differ on another important issue. Kant distinguishes between concepts of the understanding (the categories) and ideas of reason. The categories are constitutive: they are what make experience possible in the first place. The ideas—such as the soul, the world as a whole and God—are merely *regulatory*: they express the requirement for systematic unity in our cognition but correspond to nothing in experience (Grier, 2001). Kant emphatically denies that the ideas possess constitutive validity. In the appendix to the Transcendental Dialectic, he writes: “Transcendental ideas are therefore never of constitutive use … On the contrary, they have an excellent and indispensably necessary *regulatory* use, namely that of directing the understanding towards a specific goal” (Kant, 1787). Ideas are heuristic principles for the organisation of knowledge, not insights into the nature of things. In the *Critique of Judgment*, Kant explicitly applies this restriction to organisms: the principle that “nothing in an organised being is in vain” is not a constitutive law of nature, but a maxim for the reflective faculty of judgment (AA V:376).

Steiner’s response turns on the origin of ideas. If reason were merely to project a subjective need for unity onto an indifferent reality, the ideas would indeed be illusory. Steiner argues, however, that reason does not *impose* unity; rather, it *reveals* a unity that lies within the object itself: “Reason does not presuppose a specific unity, but rather the empty form of unity; it is the capacity to bring to light a harmony that lies within the object itself” (GA 2:72). The task of reason is therefore not to invent higher units, but to articulate, within the realm of concepts, the objective harmony that is already inherent in the things. Ideas arise when concepts, in their inner connectedness, are grasped as the articulated expression of this objective unity.

This is the philosophical foundation for the argument regarding organic cognition set out in Sect. 6. Kant had described the teleological unity of organisms as a purely regulative idea—as a heuristic to guide research, not as a constitutive feature of the organisms themselves (AA V:375). If Steiner is right that the ideas of reason are not subjective projections but revelations of objective unity, then the teleological idea of the organism might indeed be constitutive: not a demand we impose on nature, but the organism’s own ideal nature, grasped by reason. Whether this possibility can be realised—whether thought can indeed bring forth the specific idea of the organism from within—is the question to which we now turn.

## The cognition of inorganic nature

### From general epistemology to the forms of nature

Sections 2 to 4 have outlined the general framework of Steiner’s theory of knowledge: perception conveys one aspect of reality (sensory particulars), thinking reveals the other (ideal meaning and context), and cognition reunites what the dual organisation of the knowing subject has separated. The question now arises as to how this general framework can be applied to the specific realms of nature. In chapter 15 of *Grundlinien*, Steiner first turns to inorganic nature before moving on in the following chapter to the organic realm, where the full scope of his theory of knowledge comes to light. The present section deals with Steiner’s account of inorganic knowledge only briefly and only insofar as it serves, by contrast, to clarify what distinguishes the knowledge of organisms.

### Inorganic nature: external determination

The defining characteristic of inorganic nature is that its processes are determined by factors that are *external* to one another. An event in the inorganic realm is the result of the interaction of elements that have no internal connection: one body alters the state of another; one force modifies the effect of another. No individuality, no inner principle of form is expressed through this interaction—only the contingent convergence of independent factors (GA 2:86).^23^

The laws that explain interactions in the inorganic realm take the following form: “If this factor interacts with that factor, then this phenomenon must occur” (GA 2:92). The phenomena are *explained* when they are traced back to such fundamental relations. Thinking grasps such lawful connections between factors that exist independently of one another; it does not encounter a principle that internally generates the phenomenon itself.^24^

This form of knowledge is thus inherently subject to a crucial limitation: the concept stands *beside* perception as its complement; it does not stand *within* it as its formative principle. It is precisely here that an understanding of organic nature demands more. In an organism, the “law” does not merely regulate the interaction of already existing parts; it *generates* the parts from within. The whole precedes its parts and determines them—not as an external force, but as an immanent formative principle (which Steiner, following Goethe, termed *Typus*)^25^ In order to recognise such a being, thinking can no longer simply establish the ideal connection between given perceptions; it must *generate* the formative principle out of itself, just as the organism generates itself. This is the transition from the knowledge of the inorganic to the knowledge of the organic through “intuitive judgement” (*anschauende Urteilskraft*). It is the subject of Sect. 6.

## The cognition of organic nature: *Typus*, intuition, and *Anschauende Urteilskraft*

### Extending the concept of scientific method

The transition from inorganic to organic cognition marks a qualitative epistemological shift. In the inorganic realm, the particular element is “entirely devoid of conceptual content” (*ganz begrifflos*) and merely subsumed under an abstract general concept (GA 2:97). In the organic realm, precisely this condition fails. The individual appearance of a living being “reveals a functional, i.e. conceptual, structure. The particular bears the marks of the concept itself” (*trägt Spuren des Begriffes an sich*) (GA 2:97): its parts do not appear as contingently assembled but rather as internally articulated by a formative principle.

Kant had clearly recognised this: an organism is a being “whose intrinsic possibility emphatically presupposes the idea of a whole as that upon which the very nature and action of the parts depend” (AA V:408). He concluded, however, that our discursive understanding cannot *comprehend* such beings. We must judge organisms *as if* they were purposefully organised and *as if* they had been brought into being by a self-generating force, yet we cannot claim any genuine knowledge of this formative principle. For Kant, the teleological judgement remains merely regulative (cf. Section 1.1).

Steiner does not reject Kant’s analysis, but rather questions its premise. The error lies not in the characterisation of the organism, but in the assumption that the method of inorganic science exhausts the concept of the scientific method. Physics is not *the* scientific method; it is a *special case*, adapted to a field in which the individual is conceptless and the law stands external to the phenomenon. “The possibility that the concept of scientificity might perhaps be far more comprehensive than ‘the explanation of the world according to the laws of the physical world’—that has been forgotten” (GA 2:99).^26^

### The *Typus* as formative principle

What is required is a form of knowledge that grasps the organism *within its own framework*. In the inorganic realm, apart from the observed facts, we need only the law that links them together. In the organic realm, we need “a factor that is not passively determined by external circumstances, but which, under their influence, *actively determines itself from within*” (GA 2:102). This factor is what appears in every individual of an organism as a general formative principle. Following Goethe, Steiner calls it the *type* (*Typus*):

We shall call this general organism, following Goethe, the *Typus*. … The Typus is thus the idea of the organism: the animality in the animal, the general plant in the particular plant. (GA 2:102–103).

This is an important point, as Goethe’s *Typus* should not be confused with a fixed biological type. As Steiner wrote, the term refers to what we can also call the general concept of an organism, conceived as self-forming and dynamic.

Three features distinguish the *Typus* from the inorganic law:

Firstly, the *Typus* is “something thoroughly fluid” (*etwas durchaus Flüssiges*) (GA 2:103). It is not a fixed archetype—not a “Creator’s embodied thought”, as Agassiz claimed—but a *generative* principle that is by nature capable of transformation. This point is crucial and is easily misunderstood. The *type* is neither an abstract general concept derived by extracting common characteristics from many organisms, nor is it a Platonic form that exists unchanged in all its manifestations. The *Typus* is the formative principle *as such*—and its essence lies in *concretising itself*. Fluidity is not a deficiency (as if the concept were merely vague), but the essential character of a principle whose universality lies in its ability to generate, from within itself, an inexhaustible variety of specific forms.

This has direct implications for the relationship between the *Typus* and the theory of descent. Steiner emphasises that the *Typus* does not contradict Darwinian evolution, but rather provides what evolutionary theory cannot achieve on its own: the *inner principle* from which the specific organic character is derived. External conditions (climate, competition, available resources) determine *which* particular form of the *Typus* is realised; however, they do not bring about the organic character as such. The essential characteristics of a species “can never arise as a result of external circumstances” (GA 2:100). In Steiner’s terms, one must distinguish between the *temporally* first and the *principally* first: the simplest organism, which appeared earliest in time, is not the explanatory basis for the subsequent ones; rather, “all forms follow from the Typus—the first as well as the last—and are manifestations of the same” (GA 2:104). The *Typus* is the principle that makes the entire series of development comprehensible as an *ordered* multiplicity rather than as a random succession of forms.^27^

Secondly, the relationship between the *Typus* and its individual manifestations differs fundamentally from the relationship between a law and the phenomena it governs. In inorganic nature, “the law governs what appears as something that stands above it; the *Typus* flows into the individual living being; it identifies with it” (GA 2:106). A law of nature remains the same, regardless of which individual case it embodies; the *Typus*, on the other hand, *becomes concrete* in every organism and takes on a unique form that is inseparably linked to the formative principle itself.

Thirdly, the *Typus* determines not only the *form* but also the *content* of the particular. In inorganic nature, the senses provide the content (heat, colour, pressure), and thinking provides the lawful form. In organic nature, “content and form are closely intertwined” (GA 2:109). The *Typus* does not determine the content from the outside, but “permeates it in a living way from within, as its own” (GA 2:109).

### The developing method

This difference in subject matter necessitates a difference in the method of cognition. In inorganic science, the method is *demonstrative* (*beweisend*): under certain conditions, a specific result *must* follow; and the individual case is explained by demonstrating that it falls under the law. In organic science, this approach is not applicable. The *Typus* does not state that under certain conditions a specific result must occur; it determines “only the lawfulness of its own parts” (GA 2:108). We cannot place the *Typus* alongside the individual organism and ask whether it confirms the general rule; we must *develop* the particular form *out of* the *Typus* (GA 2:106).

Instead of the demonstrative method, organic science requires a *developing* method: “It is not that external conditions interact in a certain way and thereby produce a specific result, but that, under certain external conditions, a specific form has developed from the type” (GA 2:108).^28^

### Intuition and anschauende Urteilskraft

The developing method requires a certain cognitive ability. In inorganic cognition, thinking provides the *form* (the lawful relations) and the senses provide the *content* (the material properties of the interacting factors). In organic cognition, this separation breaks down. Since content and form are inseparable in the *Typus*, “our mind is faced with the task of participating productively in the creation of the content together with the formal element” (GA 2:109).

A mode of thinking in which content and form are given in immediate unity—as Steiner states—has always been referred to as *intuition* (GA 2:109). Steiner now clarifies what this means for the cognition of organisms: “Our power of judgment must think intuitively and intuit thinkingly” (*denkend anschauen und anschauend denken*) (GA 2:110). This is the *intuitive power of judgement* (*anschauende Urteilskraft*) which, as Steiner argues, “Goethe pointed out as a necessary form of apprehension (*notwendige Auffassungsform*) in the human spirit, which Kant claimed could not be inherent in the human being due to its entire constitution” (GA 2:110).^29^

The epistemological foundation for this claim was laid in the preceding sections. Steiner argues that thinking, in its self-transparency and its correspondence with its own content, already exhibits the very structure that is later required for organic cognition.^30^ In order to grasp the *Typus*, thought must therefore internally reproduce the formative process through which the organism generates itself—and thus produce from within the very same ideal content that the organism realises in material forma.

### The organism as self-enclosed individuality

The result of this analysis is an understanding of the organism that differs from both the mechanistic and the externally teleological perspectives. Every single organism “is an individuality that directs and determines itself from a centre. It is a self-contained whole” (GA 2:113). In inorganic nature, every single fact is only comprehensible as part of the totality of facts; the ideal of inorganic science is the complete system of natural laws. In organic science, on the other hand, the ideal consists in grasping the *Typus* in its full flexibility and tracing its concretisation through the diversity of life forms. The organism carries its principle of comprehensibility *within itself*. Steiner summarises the systematic parallel and the difference:

In organic nature, the type represents the natural law … of inorganic nature; intuition (intuitive judgment) represents demonstrative (reflective) judgement. (GA 2:110).

### The epistemic status of intuitive cognition

One objection springs to mind: can intuitive knowledge claim the same degree of epistemic certainty as demonstrative proof? Does it not reduce science to subjective inspiration or belief? Steiner addresses this concern directly. If, as the tradition since Kant maintains, one assumes that the core of reality lies in an unknowable beyond, then demonstrative proof is indeed the only substitute for the “lack of insight into the nature of things” (GA 2:112). According to such a view, any assertion that is not supported by deductive proof must appear as mere belief.

Yet Steiner’s theory of knowledge (Sections 2–4) has established a different starting point. If thinking is not a subjective process imposed upon an inaccessible reality, but rather that activity in which the core of reality *enters into experience* and determines itself in accordance with its own nature, then the criterion of scientific knowledge is not formal proof, but *insight into the thing-in-itself*. In this sense, intuition is “an immediate being-within, a penetration into the truth which gives us everything that is at all worth considering in relation to it.” Knowledge gained through intuition “is just as scientific as demonstrated insight” (GA 2:113).

This does not mean that intuitive insight is arbitrary nor infallible. Like any concept, the *Typus* must be tested against phenomena: does the formative principle, as grasped by the thinker, actually explain the specific forms that occur in experience?^31^ The developmental method is no less rigorous than the demonstrative one; it simply operates according to a different logic—the logic of *internal development* rather than *external subsumption*. The criterion of success is the same as for any insight: the reunification of perception and concept (Sect. 4.3), so that—ideally—nothing in the phenomenon remains obscure and nothing in the concept remains empty.

## Conclusion

### Summary of the argument

This paper has reconstructed the systematic argument of Steiner’s *Grundlinien einer Erkenntnistheorie der Goetheschen Weltanschauung* in five stages.

The analysis began (Sect. 2) with Steiner’s characterisation of *pure experience* as a structureless manifold—as a “world-picture” that precedes any conceptual determination and in which no relationships, no causal connections and no subject-object distinction are yet given. It was shown that this starting point, far from being naively empiricist, corresponds in essential respects to Kant’s “matter of sensation” and serves the same methodological purpose: the prevention of the introduction of untested presuppositions into the foundations of epistemology.

In the second stage (Sect. 3), thinking was defined as a *higher experience within experience*: an activity encountered within the manifold of experience, yet distinct from all other contents of experience in that it is transparent in itself and lawful from within. In thinking, the knower and the known coincide in a way that is found nowhere else in experience.

In the third stage (Sect. 4), cognition was reconceptualised as *reunification*. Perception and concept are not two independent sources of knowledge, but two sides of a single reality that have been separated from one another by the dual organisation of human cognition. Thinking reveals that aspect of reality—its meaning and its lawful interrelations—which can only appear to thinking; perception provides the sensory particulars. Cognition reunites what belongs together, thereby providing access not to a subjective construction, but to the objective nature of things—what Steiner terms *objective idealism*.

In the fourth stage (Sect. 5), this general framework was applied to *inorganic nature*. Here, the concept stands *beside* perception as its complement: inorganic cognition deals with lawful relationships between factors that stand in an external relationship to one another, and the explanation consists in grasping the necessary connections that arise from such interactions. In this realm, thought articulates the law that connects elements existing independently of one another. This marks the boundary of inorganic knowledge and prepares the transition to organic nature, where the formative principle no longer stands outside the phenomenon but operates within it.

In the fifth stage (Sect. 6), the analysis was extended to *organic nature*, where the relationship between concept and phenomenon undergoes a qualitative transformation. The formative principle of the organism—the *Typus*—does not govern its manifestations from the outside but acts within them as an immanent principle. Recognising the organism therefore requires a productive mode of thinking that can follow this self-formation from within. This is the *intuitive power of judgement*—the faculty of judgement that Goethe practised and which Steiner’s theory of knowledge seeks to ground philosophically.

### The *Grundlinien* and Kant’s challenge

The central systematic step of the *Grundlinien*, as reconstructed here, lies in Steiner’s attempt to articulate the epistemological foundations of Goethe’s scientific method. In the present work, this argument has been reconstructed throughout in systematic relation to Kant, as this is where its significance for the problem of organic cognition becomes most clearly apparent. Read in this way, Steiner’s reconstruction of Goethe leads to a position which, if successful, overcomes Kant’s restriction of teleological judgement to a purely regulative role. Kant’s restriction rests on two premises: (a) that our understanding is discursive—it can only subsume particulars under general concepts and, thus, can conceive of a whole only as being assembled from its parts; and (b) that an intuitive understanding, which would proceed from the whole to the parts, is conceivable but cannot be attributed to finite human cognition.

Steiner neither rejects Kant’s characterisation of the organism nor disputes the distinction between discursive and intuitive knowledge. His strategy is more subtle: through a careful analysis of thought itself, he demonstrates that the human mind already possesses—in the very act of thinking—those structural characteristics which Kant attributed exclusively to the *intellectus archetypus*. Thinking is *productive* (it generates its own content rather than passively receiving it), *self-transparent* (it is conscious of its own activity) and *self-determining* (its content arises from its own nature, not from external imposition). When these capacities are applied to organic nature, thinking can pursue the self-formation of the organism from within—not by projecting a purpose onto an unknowable substrate, but by internally reproducing the formative principle which the organism itself realises.

The teleological judgement, which Kant described merely as regulative, thus becomes, according to Steiner, a genuine *constitutive* judgement: the *Typus* is not a heuristic fiction, but the objective formative principle of the organism, and the intuitive insight that apprehends it is intended to convey genuine knowledge of this principle.

### Relevance for theoretical biology and philosophy of biology

The *Grundlinien* bears, avant la lettre, on five related and partially overlapping issues that remain central to contemporary philosophy of biology: (i) the epistemic status of teleology in the cognition of organisms, (ii) the concept of organic self-formation, normativity, and function, (iii) biological individuality, (iv) the processual character of living beings, and (v) the relationship between subject and object in organic epistemology.

(i) Regarding the *epistemic status of teleology in the cognition of organisms*: Contemporary interpretations of Kant fall broadly into three groups. Some retain the primarily regulative status of teleological judgement while stressing its methodological indispensability for the study of living beings (Breitenbach, 2009; Ginsborg, 2015; Nassar, 2016; Van den Berg, 2018). Others assign it a stronger, partly constitutive role in identifying organisms as specifically biological objects (Quarfood, 2006; Toepfer, 2012). A third line treats teleology as genuinely explanatory and as irreducible to mechanism, or at least as standing in a non-substitutable relation to it (Gambarotto, 2023; Walsh, 2012).

Steiner’s position cannot be placed straightforwardly within any one of these options. It is closest to the constitutive line insofar as teleology is not for him a merely optional perspective adopted in the face of explanatory difficulty; rather, it belongs to the object itself insofar as the organism is a purposively self-forming whole (Sects. 6.2–6.6). Yet Steiner also goes beyond that line, since the warrant for teleological cognition is not drawn merely from the indispensability of such judgements for biological reflection, nor primarily from a theory of explanation, but from a more basic epistemological claim: in thinking, the formative connection is given in an activity whose genesis is transparent to itself (Sects. 3.2–3.5). On this view, teleological knowledge is the appropriate mode of cognition for objects whose being consists in the generative articulation of parts from an internally determining whole. Steiner’s claim is therefore stronger than regulative readings of Kant and also more explicitly epistemological than contemporary naturalisations of teleology: what justifies teleological judgement is not first a metaphysics of natural purposes, but an account of thinking as the point at which concept and reality can be shown to belong together.

(ii) Regarding *organic self-formation, normativity, and function*, contemporary organism-centred approaches increasingly characterise living beings as self-producing and self-maintaining unities rather than as passive outcomes of externally imposed mechanisms. Some focus on autonomy, organisational closure, and the emergence of agency from the organism’s self-maintaining organisation (Di Paolo, 2005; Kauffman & Clayton, 2006; Moreno, 2018; Virenque & Mossio, 2024). Others stress the stronger claim that organisms are adaptive agents whose own activity contributes to development, function, and evolution (Barham, 2012; Jaeger, 2024; Nadolski & Moczek, 2023; Sultan et al., 2021; Uller, 2023; Walsh, 2015).

Steiner’s position intersects with these debates but is not reducible to any of them. Like contemporary theories of autonomy, he treats the organism as a self-producing whole whose parts derive their significance from the whole they serve and generate (Sects. 6.2–6.5). This also gives the problem of function and normativity a distinctive shape: what counts as a part, an activity, or a successful performance cannot be specified independently of the organismic whole in whose maintenance and articulation it participates. Like recent developmental approaches, Steiner insists that organic form is not imposed from outside but unfolds from an inner formative principle (Sect. 6.3). Yet the *Grundlinien* ground this claim in an epistemological way. Goethe’s *Typus* and developing method do not merely redescribe organisms as self-organising systems; they are meant to show how the formative principle can be followed in cognition itself, as form develops from form (Sects. 6.2–6.4). In this respect Steiner stands close to Kantian and post-Kantian attempts to understand organismic agency and purposiveness without collapsing them into mechanism (Desmond & Huneman, 2020; Gambarotto & Nahas, 2023).

(iii) Regarding *biological individuality*, contemporary philosophy of biology asks by what criteria biological individuals are to be individuated at all. Some accounts ground individuality in circular self-production and natural purposiveness, others in agency and autonomy, and still others in the organism’s capacity to establish functional boundaries (Fulda, 2023; Pradeu, 2010; Weber & Varela, 2002). Steiner’s account is relevant here because he understands the organism as an internally articulated unity whose individuality is grounded not in external delimitation but in an inner formative principle that differentiates and integrates the parts into a living whole (Sects. 5.2, 6.3, and 6.5).
(iv) Regarding the processual character of living beings, Steiner’s account is relevant insofar as the organism is understood not as a fixed bearer of properties but as a self-forming unity whose identity lies in internally articulated becoming (Sects. 6.2–6.5). This brings his position close to recent process-philosophical approaches, which argue that organisms are best understood as dynamically sustained processes rather than as stable substances (Koutroufinis & Araujo, 2023; Meincke, 2019, 2023; Nicholson & Dupré, 2018). Steiner’s contribution is therefore not a process ontology as such, but an epistemological account of how organismal becoming can be rendered intelligible.
(v) Regarding the *relationship between subject and object in biological cognition*, Hans Jonas’s philosophical biology and contemporary enactivist approaches both seek to think the organism from its inner side, whether in terms of metabolism, concern, autonomy, or sense-making (Barandiaran, 2017; Di Paolo, 2018; Jonas, 1966; Ward et al., 2017; Weber & Varela, 2002). Steiner’s answer differs in its epistemic starting point. He does not begin from the lived inwardness of the organism, but from the inner experience of thinking. For him, the inwardness of life still belongs to the side of experience or perception; only thinking is self-transparent in such a way that knower and known coincide and conceptual content becomes intelligible through its own enactment (Sects. 3.1–3.5). On this basis, the *Grundlinien* provides a philosophical foundation for a participatory knowledge of organisms such as Goethean morphology had long practised (Bortoft, 1996; Brady, 1984; Holdrege, 2013; Hueck, 2023; Riegner, 2013; Rupik, 2024).

Taken together, these issues bear directly on contemporary debates about organic development and evolution (Laland et al., 2015; Müller, 2017). Steiner’s contribution, however, lies less in proposing empirical hypotheses about these processes than in supplying an epistemological basis for understanding self-formation, function, individuality, teleology, agency, and the processuality of living beings as ontologically real aspects of organic life rather than as merely heuristic projections (Sects. 3–6).

### Organicism, vitalism, and Steiner’s later spiritual science

A final clarification concerns the relation between the organicism reconstructed here and vitalism. If by vitalism one means the postulation of a special life force alongside physical forces, neither Goethe’s method nor Steiner’s reconstruction of it depends on such a postulate. Goethe himself treated the notion of a specifically organic *Bildungstrieb* with caution, since it threatened to remain a “dark and incomprehensible point” rather than a genuine explanation (Goethe, 1987). Steiner made the same distinction explicit: Goethe, he argued, was neither a mechanist nor a vitalist. He did not explain organisms through inorganic forces alone, but neither did he invoke a special life force. What was required instead was “a mode of perception different from that through which the phenomena of inorganic nature are apprehended” (Steiner, 1897). The position at stake is therefore not a defence of neo-vitalism, but an epistemological organicism: the claim that organic form requires a mode of cognition adequate to its immanent self-formation.

At the same time, Steiner’s later spiritual science cannot simply be ignored. In his anthroposophical writings, he describes living beings in terms of an “etheric body” (*Ätherleib*) or “body of formative forces” (*Bildekräfteleib*), which can easily appear to reintroduce precisely the kind of vitalism rejected above. Within Steiner’s own conceptual framework, however, this term is not intended as a hypothetical substance inferred in order to fill an explanatory gap, but as the correlate of a transformed or intensified mode of cognition. Thus Steiner later claimed that what Goethe perceived in the plant through his intuitive, “plastic” apprehension could be called, in anthroposophical terminology, the etheric body of the plant (Steiner, 1921), and that this etheric organisation becomes accessible through an additional “organ of perception,” through which “the life of living beings itself” can be apprehended (Steiner, 1904; cf. Hueck, 2025a). Whether this later extension is philosophically defensible lies beyond the scope of the present article. What matters here is the continuity of the underlying epistemological structure: Steiner’s later spiritual-scientific claims deepen the account of intuitive organic cognition reconstructed in the *Grundlinien*, rather than simply replacing it with an external vital force.

### Goethe’s method as participatory science

The deeper significance of the *Grundlinien* lies in the original task from which the work arose: to interpret Goethe’s method as a genuine mode of scientific cognition. On Steiner’s reading, Goethe’s morphology points toward a participatory science in which the knower must actively enter into the formative articulation of the phenomenon itself. The present argument suggests that this approach deserves renewed attention for systematic reasons. Since biology is compelled anew to think in terms of self-formation, purposiveness, normativity, individuality, agency, and the temporally unfolding identity of living beings, the question re-emerges whether a strictly external mode of cognition is sufficient to its object. In this respect, the *Grundlinien* proposes a way of understanding organisms as internally articulated, self-forming unities without collapsing their intelligibility either into mere heuristic projection or into reductionist dissolution.

## Footnotes

1 I use the following abbreviations: AA = Akademie Ausgabe of Kant’s collected works, volume and page number; FA = Frankfurter Ausgabe of Goethe’s Schriften; GA = Rudolf Steiner Gesamtausgabe (complete works) with number of volume. All translations from German texts (Steiner, Goethe, Volkelt) are my own. The GA can be accessed online under www.rudolf-steiner-online.de, which can be translated with an online translation program.

2 Since Steiner also expounds on the same ideas in *Wahrheit und Wissenschaft* (Steiner, 1892), and in his major philosophical work *Die Philosophie der Freiheit* (Steiner, 1894), in some cases in greater detail or with greater precision, I shall also quote from these books where appropriate. The present article reconstructs Steiner’s interpretation of Goethe’s scientific method, not Goethe’s scientific thought as such. For broader discussions of Goethe’s morphology, intuitive judgment, and empirical method, see Förster (2012, ch. 11), Nassar (2022), Goy (2019), and Hueck (2025c), where I engage more extensively with the relevant Goethe scholarship.

3 A later parallel is Husserl’s call to return “to the things themselves” and to suspend unchecked presuppositions (Husserl, 1901, p. 7); the similarity, however, is of a methodological rather than a dogmatic nature.

4 Steiner explicitly clarifies this point in *Wahrheit und Wissenschaft*: The immediately given, he writes, is never actually present to human beings in this pure form, since there is nowhere a boundary between the passive orientation towards the immediately given and its cognitive apprehension. Even “the separation of individual details from the completely undifferentiated given world-picture is already an act of thought.” The boundary between the given and the known, he concludes, “must be drawn artificially” (Steiner, 1892). In this sense, *pure experience* functions not as a psychological datum, but as a methodological concept of delimitation. This distinguishes Steiner’s use of the term from William James’s later radical empiricism, for which relations are themselves directly experienced and must therefore be counted as real elements of experience (James, 1912). For Steiner, by contrast, even the grasping of something *as* a relation already presupposes thinking; relations therefore cannot belong to pure experience in his technical sense.

5 Steiner’s examples—a tree, a table—are, of course, already conceptually constituted objects, not objects of “pure experience” in the sense of Sect. 2.2. The shift in level is intentional: Steiner shows here that even at the level of ordinary, conceptually structured experience, the predicate “subjective” is nowhere to be found in what is given (GA 2:38). It is introduced through philosophical reflection, not discovered in the phenomena.

6 Johannes Müller’s theory of specific sensory energies and Helmholtz’s sensory physiology promoted the view that perceived properties depend on the organisation of the sensory apparatus (Hatfield, 1991).

7 Steiner agrees with Sellars that an unstructured given cannot by itself amount to cognition. Unlike Sellars, however, he holds that thinking itself can be encountered within experience and thereby investigated phenomenologically. Like McDowell, Steiner denies that human beings ever stand before a wholly unconceptualized given in any simple sense (see above, Sect. 2.2). Yet he insists that in thinking the conceptual contribution becomes explicit and self-transparent in a way that it does not in ordinary perception.

8 That thinking depends on the thinker’s own activity is, Steiner later argues, confirmed by the simple fact that we can *abstain* from it (Steiner, 1894). Every other element of experience—a colour, a sound, a feeling—presents itself, as soon as the senses are directed towards it, as a *given* whose specific content is not produced by the subject; a thought, on the other hand, must be actively sustained and ceases when the activity is withdrawn.

9 “I must work through the thought, must recreate its content, must experience it inwardly down to its very smallest details, if it is to have any meaning for me at all” (GA 2:47). And in *Die Philosophie der Freiheit*: “One must not confuse ‘having mental images’ with processing thoughts through thinking. (…) [R]eal thinking must always be intentional. … what matters is that nothing is intended which, whilst taking place, does not appear to the ‘I’ entirely as its own activity, one that it can comprehend “ (Steiner, 1894).

10 The structural parallel to Husserl’s concept of *Evidenz*—the self-given nature of the intended object in the act of intending—is striking, although Steiner’s analysis predates Husserl’s *Logische Untersuchungen* (Husserl, 1900, 1901) by more than a decade. Whether there is a direct historical influence remains an open question that goes beyond the scope of this paper.

11 The philosophical-historical lineage of this position—from Aristotle’s account of *nous* as “identical in act” with its object (Aristotle, 2017, p. III,4 430a1-5) via Fichte’s *Tathandlung*, Hegel’s dialectical self-unfolding of the Idea and Frege’s contemporaneous anti-psychologism—deserves a more comprehensive treatment than this essay can provide. The distinctive character of Steiner’s contribution—the claim that active production and objective self-determination coincide in the experience of thinking—and its relationship to the occasionalist resonance of his key concept *Gelegenheitsursache* will be addressed in a separate study.

12 The Kantian notion that comes closest to Steiner’s conception of the self-transparency of thinking is Kant’s conception of the a priori construction of concepts in pure intuition, particularly in mathematics and geometry (Carson, 1997; Friedman, 2012; Kant, 1787). In Kant’s view, necessity is revealed in such a construction because the concept is represented in the a priori forms of intuition, space and time. Kant’s focus is therefore on the constructed object and on the necessity expressed therein, whereas Steiner makes the self-generating activity of thinking the decisive epistemological datum. A related point emerges in *Die Philosophie der Freiheit*, where Steiner observes that the thinker tends to “forget” thinking whilst engaging in it: “It is not the act of thinking that preoccupies him, but the object of thought that he is considering” (Steiner, 1894). If the activity of thinking itself is to become an object of observation, Steiner argues, this requires a “state of exception” (*Ausnahmezustand*) (Steiner, 1894). His argument in *Grundlinien*, however, is not that one must observe the emergence of thinking simultaneously within the act of thinking itself, but rather that the self-creating character of thinking is philosophically decisive.

13 Kant was criticised by Reinhold, Fichte and later Hegel for failing to fully ground the objective validity of thinking presupposed by the critical project. As Eckart Förster’s reconstruction suggests, these thinkers did not primarily reject Kant’s insights; rather, they argued that critical philosophy had not yet fully justified its own premises and thus had not fully justified the objective validity of thinking (Förster, 2012, pp. 153–168,179–181). Steiner’s argument can be read as an attempt to meet this demand by demonstrating, on the basis of the self-transparent character of thinking, why the lawful forms of thought are not merely subjective.

14 A structurally similar objection was raised against reflexive theories of knowledge in post-Kantian thought. Henrich, for example, argues that all “models of reflection” of self-consciousness are circular, since the act of reflection must already possess what it purports to establish (Henrich, 1967, p. 14).

15 The question is not new. It is, in various forms, the problem on which both Frege’s and Hegel’s theories of thinking ultimately founder: Frege’s situating of thoughts in a “third realm” leaves unresolved how an embodied mind can gain access to non-spatial entities (Burge, 1992; Frege, 1918–1923); Hegel’s dialectical self-unfolding of the Idea has been criticised for failing to show how it relates to the world of sensory experience (Pippin, 2012; Stern, 2009). Steiner’s response takes a different path: not by postulating a separate realm (Frege) or by deriving the sensible from the logical (Hegel), but by demonstrating that thought and perception are *two sides of one and the same reality*.

16 The full philosophical implications of Steiner’s conception of thinking as an “organ of apprehension” (*Organ des Auffassens*)—through which what would otherwise remain a blind juxtaposition is grasped as a necessary connection—cannot be pursued here. Its relationship to Aristotle’s *nous*, Fichte’s *Tathandlung*, Frege’s anti-psychologism and certain contemporary enactivist approaches will be elaborated in a separate study.

17 For an example see the description of Goethe’s idea of plant metamorphosis as a dynamic process of threefold expansion and contraction with progression of a common generative principle (Goethe’s “leaf”) in (Hueck, 2025c).

18 The structural parallel to Aristotle’s hylomorphism is obvious, but it also points to an important difference: For Aristotle, the distinction between matter (*hyle*) and form (*eidos*) is *ontological*—it belongs to the constitution of things themselves (Pfeiffer, 2021; Rapp, 2021). For Steiner, the distinction is *epistemological*: it arises from the organisation of the knowing subject. The act of cognition does not connect two ontologically distinct principles but overcomes an epistemologically conditioned separation.

19 In *Die Philosophie der Freiheit*, Steiner illustrates this with a simple but instructive example. If I receive a rosebud today, the image presented to me by my perception initially appears as a self-contained whole. If, however, I place the bud in water, I shall receive a completely different image of my object tomorrow. “It is entirely arbitrary to regard the sum of what we learn about a thing through mere perception as a totality, as a whole, and to treat what emerges through thinking contemplation as something foreign that has nothing to do with the thing itself” (Steiner, 1894). The perceptual image at a given moment is “merely a random cross-section of an object that is in a state of constant becoming” (Steiner, 1894). What thinking contributes—the law of development that links the bud to the flower—is not a subjective addition, but the completion of a reality that perception, by its very nature, can only provide in momentary fragments.

20 See footnote 16 on the idea as an “organ of apprehension”.

21 Steiner’s account of this inner coherence owes much to Hegel’s Science of Logic (cf. Pippin, 2012), although the difference remains methodological in nature: Hegel derives the self-movement of the concept dialectically; Steiner, on the other hand, insists that it must be grasped in the self-transparent execution of thought, where the thinker provides the occasion for the appearance of the content of thinking, but not the law that determines it.

22 The idea of the organism, for example, does not function in exactly the same way in biology as in social thought, even though in both cases it denotes an internally ordered whole whose parts can only be understood through their relationship to one another.

23 Steiner’s characterisation of inorganic nature through purely external determination mirrors Kant’s systematic exposition in the *Metaphysical Foundations of Natural Science* (1786), in which matter is defined by external relations: as “that which moves in space”, determined by forces of attraction and repulsion acting between individual bodies, as well as by the mechanical transmission of motion (AA IV:543). The contrast with the organism—which, as Kant argues in the *Critique of Judgment*, can only be understood as a self-generating being (Kant, 1790)—is thereby heightened: the organism is precisely that which *cannot* be grasped by the categories of external determination that suffice for inorganic nature.

24 In Part I of the *Einleitungen zu Goethes naturwissenschaftlichen Schriften* (1884), Steiner characterises the understanding of inorganic nature as the grasping of sensually real processes in a common concept that expresses their lawful relationship. Cause and effect are brought together in thought, yet the concept itself does not generate or determine the process; it serves the mind as a means of uniting what is given in the sensory world (Steiner, 1987).

25 In the *Einleitungen*, Steiner also referred to this principle as the “entelechy” of the organism: “Entelechy is the force that calls itself into being from within itself” (Steiner, 1987).

26 Steiner directs this criticism not only at Kant, but also at the mechanistic biology of his own time, in particular at Haeckel’s demand that “the causal connection between phenomena must be asserted everywhere” and that, with sufficient knowledge, all mental functions could be reduced to “a mathematical formula of the soul” (GA 2:100). The same criticism applies, mutatis mutandis, to contemporary reductionist programs that seek to explain organisms entirely through molecular mechanisms.

27 Goethe’s *Urpflanze* is the paradigmatic example: it is not a specific plant that existed first and from which all others are derived, but the formative principle that manifests itself *in* all plants and can be traced *through* their metamorphic variations. As Goethe traced the transformation of the leaf through sepal, petal, stamen and carpel, he followed this fluid principle as it developed into specific organs. See (Hueck, 2025c) for a detailed analysis.

28 The developing method corresponds to what Goethe practised in his morphological studies: the tracing of leaf forms through the metamorphic series or the derivation of the vertebrate skull from the general vertebrate type. In any case, the method does not subsume details under a fixed law but *follows* the formative principle as it takes concrete form. See (Hueck, 2025c) for a detailed analysis of Goethe’s morphological practice in this sense.

29 The reference is to Kant’s §77 of the *Critique of Judgement*, where he introduces the concept of an *intuitive understanding* (*intuitiver Verstand*) or *intellectus archetypus*, which would proceed from the whole to the parts – grasping the universal as that which determines the particular – in contrast to our discursive understanding (AA V:407). Kant emphasises that such an understanding, although conceivable, cannot be attributed to finite human cognition. For a detailed discussion of Kant’s §77 and its significance for Goethe’s concept of the organism, see (Förster, 2009, 2012, pp.138–152).

30 As Jennifer Mensch has shown, Kant’s critical philosophy itself is deeply influenced by organic and epigenetic models of development: The faculty of understanding is not understood as a mere, receptive receptacle of forms, but as a spontaneous force whose a priori structures are conceived in terms of an inner genesis, culminating in Kant’s description of transcendental idealism as a “system of the epigenesis of pure reason” (Mensch, 2013; Kant, 1787; see also Genova, 1974; Ypi, 2021). Kant also characterises the systematic unity of reason in organic terms, as a whole whose parts develop from a common germ (Kant, 1787). In this sense, Kant already has a conception of an inner kinship between the structure of cognition and the structure of the organic. However, he does not conclude that human cognition can attain the kind of intuitive understanding required for the cognition of organisms; the affinity remains limited by the discursive character of the human mind. Steiner’s claim is stronger: in the self-knowledge of thought, he argues, the productive and self-articulating structure—which Kant could only approach indirectly—is already present in experience itself.

31 For example, does it provide compelling insight into the similarities and differences between plants and animals (Hueck, 2025b)?
